# Increase of TREM2 during Aging of an Alzheimer’s Disease Mouse Model Is Paralleled by Microglial Activation and Amyloidosis

**DOI:** 10.3389/fnagi.2017.00008

**Published:** 2017-01-31

**Authors:** Matthias Brendel, Gernot Kleinberger, Federico Probst, Anna Jaworska, Felix Overhoff, Tanja Blume, Nathalie L. Albert, Janette Carlsen, Simon Lindner, Franz Josef Gildehaus, Laurence Ozmen, Marc Suárez-Calvet, Peter Bartenstein, Karlheinz Baumann, Michael Ewers, Jochen Herms, Christian Haass, Axel Rominger

**Affiliations:** ^1^Department of Nuclear Medicine, Ludwig-Maximilians-Universität MünchenMunich, Germany; ^2^Department of Biochemistry, Biomedical Center (BMC), Ludwig-Maximilians-Universität MünchenMunich, Germany; ^3^Munich Cluster for Systems Neurology (SyNergy), Ludwig-Maximilians-Universität MünchenMunich, Germany; ^4^DZNE—German Center for Neurodegenerative DiseasesMunich, Germany; ^5^Laboratory of Neurodegeneration, International Institute of Molecular and Cell BiologyWarsaw, Poland; ^6^Roche Pharma Research and Early Development, Neuroscience Discovery, Roche Innovation Center Basel, F. Hoffmann-La Roche Ltd.Basel, Switzerland

**Keywords:** Alzheimer’s disease, neuroinflammation, TREM2, amyloid-PET, TSPO-PET

## Abstract

Heterozygous missense mutations in the triggering receptor expressed on myeloid cells 2 (TREM2) have been reported to significantly increase the risk of developing Alzheimer’s disease (AD). Since TREM2 is specifically expressed by microglia in the brain, we hypothesized that soluble TREM2 (sTREM2) levels may increase together with *in vivo* biomarkers of microglial activity and amyloidosis in an AD mouse model as assessed by small animal positron-emission-tomography (μPET). In this cross-sectional study, we examined a strong amyloid mouse model (PS2APP) of four age groups by μPET with [^18^F]-GE180 (glial activation) and [^18^F]-florbetaben (amyloidosis), followed by measurement of sTREM2 levels and amyloid levels in the brain. Pathology affected brain regions were compared between tracers (dice similarity coefficients) and pseudo-longitudinally. μPET results of both tracers were correlated with terminal TREM2 levels. The brain sTREM2 levels strongly increased with age of PS2APP mice (5 vs. 16 months: +211%, *p* < 0.001), and correlated highly with μPET signals of microglial activity (*R* = 0.89, *p* < 0.001) and amyloidosis (*R* = 0.92, *p* < 0.001). Dual μPET enabled regional mapping of glial activation and amyloidosis in the mouse brain, which progressed concertedly leading to a high overlap in aged PS2APP mice (dice similarity 67%). Together, these results substantiate the use of *in vivo* μPET measurements in conjunction with post mortem sTREM2 in future anti-inflammatory treatment trials. Taking human data into account sTREM2 may increase during active amyloid deposition.

## Introduction

Alzheimer’s disease (AD), the most common cause of dementia, has an exponentially increasing incidence as a function of age, and is thus imposing an ever more onerous burden on health care in countries with aging populations (Ziegler-Graham et al., 2008). This motivates a worldwide effort to find new biomarkers predicting future cognitive decline in patients with suspicion of early AD, and likewise for use as outcome measures in clinical trials of innovative disease-modifying agents (Weiner et al., 2012). While the classical hallmarks of AD pathology are the accumulation of extracellular amyloid plaques and intracellular neurofibrillary tangles (Duyckaerts et al., 2009), there is increasing evidence that microglia and neuroinflammation is related directly to the pathogenesis of the disease (Heneka et al., 2015). Many aspects of the amyloid and tau pathologies are closely recapitulated in transgenic rodent models for AD (Teipel et al., 2011; Hall and Roberson, 2012), which can be monitored by small animal positron-emission-tomography (μPET) using radioligands targeting the proteins accumulating in AD (Virdee et al., 2012; Zimmer et al., 2014). In particular, imaging of the accumulation of the β-amyloid peptide (Aβ) has been proven useful for the *in vivo* detection of AD pathology in patients (Price et al., 2005), and this class of tracers are currently used for Aβ imaging in transgenic mice (Manook et al., 2012; Rominger et al., 2013). The availability of different *in vivo* tracers (i.e., for amyloid, glial activation) may allow us to study the sequence of events that occur in these mouse models.

Microglia cells are the resident macrophages of the brain. Two independent studies recently identified rare heterozygous variants in the triggering receptor expressed on myeloid cells 2 (TREM2), which increase the risk for late onset AD significantly (Guerreiro et al., 2013; Jonsson et al., 2013). TREM2 undergoes regulated intramembrane proteolysis (Wunderlich et al., 2013; Kleinberger et al., 2014) releasing the soluble TREM2 (sTREM2) which can be readily detected in biological fluids, such as the cerebrospinal fluid (CSF; Piccio et al., 2008; Kleinberger et al., 2014). Recent studies indicate a dynamic increase in CSF sTREM2 levels across the continuum of AD, with a significant increase already present at the early symptomatic stages (Suárez-Calvet et al., 2016b). Interestingly, sTREM2 levels show a strong correlation with markers of neuronal degeneration, probably due to a change of the glia reactivity in response to neuronal injury (Heslegrave et al., 2016; Piccio et al., 2016; Suárez-Calvet et al., 2016b). It follows that sTREM2 levels might serve as a potential biomarker for microglial activation during AD pathogenesis.

Activated microglia express high levels of the 18-kDA translocator protein (TSPO), which is known as a target detectable with a variety of PET ligands, among which [^18^F]-GE180 has excellent properties for quantitation of TSPO (Dickens et al., 2014; Wickstrøm et al., 2014). Dual tracer μPET imaging of Aβ and TSPO was recently established in the transgenic PS2APP mouse model (Brendel et al., 2016), a strain that is known for a strong inflammatory component besides the remarkable age-dependent amyloidosis (Richards et al., 2003; Ozmen et al., 2009); the dual tracer approach revealed associations of neuroinflammation and amyloidosis. TSPO μPET with [^18^F]-GE180 as well successfully detected age-dependent neuroinflammation in APP/PS1dE9, another AD mouse model, and to a lesser extent in wild-type (WT) mice (Liu et al., 2015) and [^11^C]PBR28 showed increased uptake in 5XFAD mice compared to age-matched WT mice (Mirzaei et al., 2016).

Given this background, the aims of the present cross-sectional multitracer study were: (1) to study the age-dependent changes in sTREM2 levels and μPET signals for the second generation TSPO tracer [^18^F]-GE180 together with that of the Aβ tracer [^18^F]-florbetaben in PS2APP and WT mice; (2) to evaluate the association of sTREM2 levels in the brain with the μPET signals and Aβ levels; and (3) to define the spatial and temporal relationships of TSPO activity and amyloidosis during aging of these mice.

## Materials and Methods

### Radiochemistry

Radiosynthesis of [^18^F]-GE180 was performed as previously described (Wickstrøm et al., 2014), with slight modifications (Brendel et al., 2016), a procedure yielding radiochemical purity exceeding 98%, and specific activity of 1400 ± 500 GBq/μmol at end of synthesis. Radiosynthesis of [^18^F]-florbetaben was performed as previously described (Rominger et al., 2013), a procedure yielding radiochemical purity exceeding 98%, and specific activity of 80 ± 20 GBq/μmol at the end of synthesis.

### Animals

All experiments were carried out in compliance with the National Guidelines for Animal Protection, Germany with the approval of the regional Animal care committee of the Government of Oberbayern (Regierung Oberbayern), and were overseen by a veterinarian. Female animals were housed in a temperature- and humidity-controlled environment with a 12-h light–dark cycle, with free access to food (Ssniff, Soest, Germany) and water.

The transgenic B6.PS2APP (line B6.152H) is homozygous for both, the human presenilin (PS) 2, N141I mutation and the human amyloid precursor protein (APP) K670N, M671L mutation. The APP and PS2 transgenes are driven by mouse Thy-1 and mouse prion promoters, respectively. This line had been created by co-injection of both transgenes into C57Bl/6 zygotes (Richards et al., 2003). Homozygous B6.PS2APP mice show first appearance of plaques in the cerebral cortex and hippocampus at 5–6 months of age (Ozmen et al., 2009).

### Study Design

μPET with [^18^F]-GE180 and [^18^F]-florbetaben emission recordings (maximum 10 days apart) were obtained cross-sectionally in equal-sized groups (*N* = 8) of PS2APP and WT mice aged 5, 8, 13, and 16 months (±0.5 months). Group-size calculations were planned based on the assumption of a type I error *α* = 0.05, giving a power of 0.8, and with the possibility of substitution in case of dropouts. Within 1 week after completion of the final PET session, PS2APP mice were perfused with PBS while deeply anesthetized, and brains were removed for biochemical and immunohistochemical analyses. In case of dropouts (*N* = 7) additional age-matched mice were substituted for the dual μPET ([^18^F]-GE180 and [^18^F]-florbetaben) arm of the study, as equal group sizes are necessary for optimal statistical parametric mapping (SPM) analysis. For biochemical and immunohistochemical analyses in WT mice groups of *N* = 6 mice at 6 and 18 months of age were used without prior PET. A detailed overview of the different groups of mice and all modalities is provided in Table 1.

**Table 1:** **Study overview for the different assessed modalities**.

Mouse Model	Age (months)	[^18^F]-GE180 μPET (N)	[^18^F]-florbetaben μPET (N)	sTREM2/Aβ/ cytokines (N)	Immuno-histochemistry (N)
PS2APP	5	8	8	6	6
	8	8	8	4	4
	13	8	8	8	8
	16	8	8	7	7
C57Bl/6	5	8	8		
	6			6	6
	8	8	8		
	13	8	8		
	16	8	8		
	18			6	6

### μPET

#### μPET Data Acquisition and Reconstruction

All mice were anesthetized with isoflurane (1.5%, delivered at 3.5 l/min) and placed in the aperture of the Siemens Inveon DPET, as described previously (Rominger et al., 2010). For static μPET recordings, anesthesia was maintained between tracer injections until conclusion of the μPET acquisition, so as to ensure comparable physiological conditions for the different tracers.

[^18^F]-GE180 μPET: PS2APP mice and age-matched C57Bl/6 controls were scanned in a full dynamic μPET setting: Upon injection to a tail vein of 8.8 ± 2.3 MBq [^18^F]-GE180 (in 150 μl saline), a 90 min emission recording was initiated, followed by a 15 min transmission scan using a rotating [^57^Co] point source. The 60-90 min static single frame recordings were reconstructed based on prior knowledge from our methodological investigation that this time window serves best for standard-uptake-value-ratios (SUVR) quantitation (Brendel et al., 2016).

[^18^F]-florbetaben μPET: Static single frame emission recordings were made in the interval 30–60 min after injection to a tail vein of 9.9 ± 1.7 MBq [^18^F]-florbetaben (in 150 μl saline), followed by a 15 min transmission scan made using a rotating [^57^Co] point source.

For all μPET recordings, reconstruction was performed with four ordered-subset-expectation-maximization 3D iterations and 32 maximum-a-posteriori 3D iterations, a zoom factor of 1.0, scatter-, attenuation- and decay-corrected, leading to a final voxel dimension of 0.78 mm × 0.78 mm × 0.8 mm.

#### Image Co-Registration and Quantitative μPET Data Analyses

Static datasets (30–60 or 60–90 min) were co-registered to an MRI mouse atlas (Dorr et al., 2007) by a manual rigid-body transformation (TX_rigid_) using the PMOD fusion tool (V3.5, PMOD Technologies Ltd.), after blinding the mouse identity to the reader. Accurate initial alignment was verified by a second experienced reader. In the second step, a reader-independent fine co-registration to tracer-specific templates was performed. Templates were generated by averaging all age specific PET scans for a single tracer, with minor spatial re-registrations to guarantee optimal overlapping of the two PET templates. Here, the initial manual μPET-to-MRI atlas fusion images were normalized by non-linear brain normalization (TX_BN_) to the tracer-specific templates using the PMOD brain normalization tool (equal modality; smoothing by 0.6 mm; nonlinear warping; 16 iterations; frequency cutoff 3; regularization 1.0; no thresholding). The concatenation of TX_rigid_ and TX_BN_ was then applied to μPET frames in the native space, so as to obtain optimal resampling with a minimum of interpolation.

Based on our previous experience, [^18^F]-GE180 and [^18^F]-florbetaben data were scaled by different white matter reference regions. In the case of [^18^F]-GE180 we used a 29 mm^3^ white matter volume edited from the cerebellum and the brainstem (Brendel et al., 2016), whereas for [^18^F]-florbetaben: we used a 67 mm^3^ volume comprising pons, midbrain and hindbrain parts of the subcortical white matter (Overhoff et al., 2016). A main forebrain target volume-of-interest (VOI; 156 mm^3^) comprising most of the frontal and parietal cortices as well as the hippocampus and the thalamus was used for both tracers to generate [^18^F]-GE180 and [^18^F]-florbetaben (SUVR_CTX/WM_), which constitute the quantitative μPET endpoints.

#### SPM Analysis and Dice Coefficients

For both tracers, whole-brain voxel-wise comparisons of white matter scaled images between age matched groups of TG vs. WT mice (each *N* = 8) were performed by SPM using SPM5 routines (Wellcome Department of Cognitive Neurology, London, UK). This analysis was implemented in MATLAB (version 7.1), as adapted from Sawiak et al. (2009) for mouse data. T-Score maps were all corrected for multiple comparisons (FDR-corrected) at a significance level of *p* < 0.05. Respective FDR-corrected T-score thresholds were used for binarization (1 = significantly different to WT/0 = not significantly different to WT) of the maps for each tracer. For each age group, combined tracer distribution maps were generated with four categories in each voxel: 0 = not significantly elevated for [^18^F]-GE180 and [^18^F]-florbetaben; 1 = significantly elevated for [^18^F]-GE180 but not [^18^F]-florbetaben; 2 = significantly elevated for [^18^F]-florbetaben but not [^18^F]-GE180; 3 = significantly elevated for both [^18^F]-GE180 and [^18^F]-florbetaben. These distributions were consecutively compared relative to the whole brain volume (%-occupancy), according to the distributions of each tracer (stand-alone vs. combined voxels). Dice coefficients (Förster et al., 2012) between non-binarized T-score maps of [^18^F]-GE180 and [^18^F]-florbetaben were calculated for each time point to assess the agreement of both tracer alterations as well with regard to their regional magnitude.

### Brain Extraction and Immunoblot Analysis

Frozen mouse cerebrum was cryo-pulverized in liquid nitrogen and sequentially extracted in Tris-buffered saline (TBS) followed by extraction with TBS-Triton (1%). In brief, 30 mg cryo-pulverized samples of brain tissue were homogenized in ice-cold TBS (10w/v) using a 26G needle and cleared by ultracentrifugation (186,000× g, 1 h, 4°C). The resulting pellet was washed once with TBS, extracted with TBS-Triton (TBS-T fraction) and lysate cleared again by ultracentrifugation (186,000× g, 1 h, 4°C).

For immunoblot analysis cryo-pulverized brains were extracted in immunoprecipitation lysis buffer (25 mM Tris-HCl pH 7.4, 150 mM NaCl, 1% NP-40, 1 mM EDTA, 5% glycerol) and cleared by centrifugation (17,000× g, 30 min, 4°C). Protein concentrations were measured using the bicinchoninic acid (BCA) method (Pierce). TREM2 was immunoprecipitated from 300 μg brain lysates using biotinylated anti-TREM2 (BAF1729; R&D Systems) and Streptavidin sepharose (GE Healthcare) overnight at 4°C. Streptavidin sepharose was washed three times with PBS and proteins eluted by boiling in 2× Laemmli sample buffer supplemented with beta mercaptoethanol for 10 min at 95°C.

Proteins were separated by SDS-PAGE and transferred either onto polyvinylidene difluoride membranes (Hybond P; Amersham Biosciences) or Nitrocellulose membranes (GE Healthcare) and probed with rat anti-mouse TREM2 (clone 5F4; Xiang et al., 2016), rabbit anti-GFAP (Dako), rabbit anti-IBA1 (Wako), rabbit anti-TSPO (Abcam) and anti-GAPDH (Sigma). Bound antibodies were visualized by corresponding HRP-conjugated secondary antibodies using enhanced chemiluminescence technique (Pierce). All buffers were supplemented with protease inhibitors (Sigma).

### Quantification of sTREM2 and Cytokines by ELISA

sTREM2 and cytokines (MSD proinflammatory panel) were quantified in TBS fractions from mouse brain using the Mesoscale platform similar to previously described methods (Kleinberger et al., 2014). The MSD proinflammatory panel was performed according to manufacturer’s recommendations. For sTREM2, streptavidin-coated 96-well small-spot plates were blocked overnight at 4°C in blocking buffer (3% bovine serum albumin and 0.05% Tween-20 in PBS, pH 7.4). For the detection of mouse sTREM2, plates were incubated for 1 h at RT with 0.25 μg/ml biotinylated polyclonal goat anti-mouse TREM2 capture antibody (R&D Systems; BAF1729) diluted in sample diluent (1% bovine serum albumin and 0.05% Tween-20 in PBS, pH 7.4). Plates were washed subsequently for four times with washing buffer (0.05% Tween-20 in PBS) and incubated for 2 h at RT with samples diluted 1:2 in assay buffer supplemented with protease inhibitors (Sigma). A recombinant mouse TREM2 protein (Hölzel Diagnostika) was diluted in assay buffer in a two-fold serial dilution and used for the standard curve (Concentration range: 2000–31.25 pg/ml). Plates were washed three times with washing buffer before incubation for 1 h at RT with 1 μg/ml rat monoclonal anti-TREM2 antibody (R&D Systems, MAB1729) diluted in assay diluent. After three additional washing steps, plates were incubated with 0.5 μg/ml SULFO-TAG-labeled anti-rat secondary antibody (Meso Scale Discovery) and incubated for 1 h at RT. Lastly, plates were washed three times with wash buffer followed and developed by adding 1X Meso Scale Discovery Read buffer. The light emission at 620 nm after electrochemical stimulation was measured using the Meso Scale Discovery Sector Imager 2400 reader. Calculation of the concentration of sTREM2 was performed with the MSD Discovery Workbench v4 software (MSD).

### Total Aβ ELISA

Total Aβ was quantified in TBS-Triton fractions similarly as described above using biotinylated 2D8 as capture antibody, mouse monoclonal 4G8 as detection antibody and SULF-TAG-labeled anti-mouse secondary antibodies. A recombinant human Aβ1–40 peptide (MSD) was diluted in assay buffer supplemented with 6% fetal calf serum in a three-fold serial dilution and used for the standard curve (Concentration range: 33–46 pg/ml).

### Immunohistochemistry: Acquisition and Image Analysis

Cerebral hemispheres intended for immunohistochemistry (random side) were fixed by immersion in 4% paraformaldehyde at 4°C for 24 h. Two representative 50 μm thick slices per animal were then cut in the axial plane using a vibratome (VT 1000 S, Leica, Wetzlar, Germany). Free-floating sections were permeabilized with 2% Triton X-100 overnight and blocked with 10% normal goat serum. We obtained immunohistochemical labeling of microglia using an Iba1 primary antibody (dilution; Wako, 1:200), and the A-21244 secondary antibody (Invitrogen, 1:500). The TSPO staining was obtained by incubation with anti-PBR antibody (Abcam, 1:300) and the A-11034 secondary antibody (Invitrogen, 1:500). Fibrillary Aβ plaques were stained with the fluorescent dye methoxy-X04 (0.01 mg ml^−1^ in phosphate-buffered saline at pH 7.4 for 15 min) as described previously (Brendel et al., 2015b). The unbound dye was removed by three washing steps with PBS, and the slices were then mounted on microscope slides with fluorescent mounting medium (Dako, Germany). Images were acquired on an epi-fluorescence microscope (Axio Imager.M2 with ApoTome.2, Jena, Zeiss, Germany). Imaging of the whole slice was performed in tile scan mode, which allows automatic stitching of an array of fields of view. The area and number of plaques and microglia were automatically counted using Imaris software (Imaris 7.6.5; Bitplane, Zurich) in the entire cerebral cortex. These analyses were performed by an operator who was blind to the μPET results.

### Statistics

Univariate analysis of variance (ANOVA) with Tukey *post hoc* test was performed to compare values of different age groups in PS2APP mice or to compare PS2APP mice against WT littermates. Student’s *t*-test was used to compare results in groups of young and aged WT. Normal distribution of data was verified by the Kolmogorov-Smirnov test. For VOI-based correlation analyses of μPET-derived estimates for microglial activation and amyloidosis with sTREM2, Pearson’s coefficients of correlation (R) were calculated. Multiple regression analysis was performed to investigate the association of μPET-derived estimates for microglial activation and amyloidosis with sTREM2 levels with age as a fixed effect. The standardized regression coefficients (β) are reported. IBM SPSS Statistics (version 22.0; SPSS, Chicago, IL, USA) was used for all statistical tests. A threshold of *p* < 0.05 was considered to be significant for rejection of the null hypothesis. Standardized differences between PS2APP and WT mice were computed for the defined time points in PS2APP mice (under the assumption that measures in WT with only two time-points are developing linearly during aging) and divided by the standard deviation. For TBS Aβ levels at 5 and 8 months of age, a combined standard deviation was used as an approximation for both time points as the singular results were obviously distorted by the low standard deviation of mice aged 5 months.

## Results

### Progressive Age-Dependent Increase of sTREM2 Levels in the Brain of WT and PS2APP Mice

**Figure 1:**
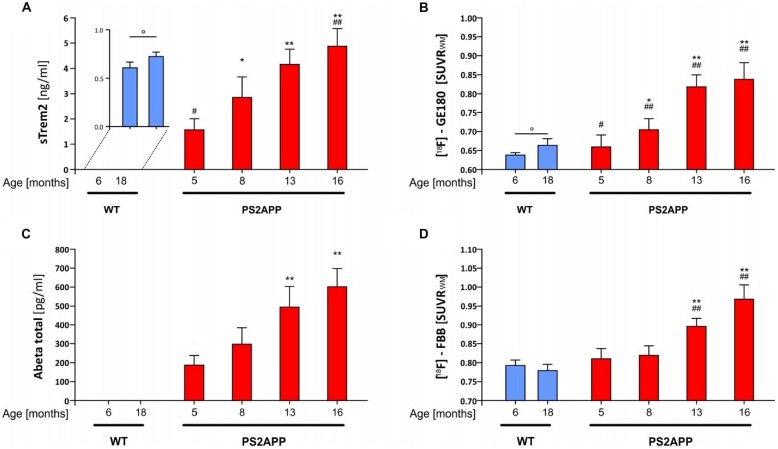
**Soluble triggering receptor expressed on myeloid cells 2 (sTREM2) increases in an age dependent manner in brains of PS2APP mice. (A)** *Post mortem* levels of sTREM2 were quantified by ELISA from forebrain tissue at indicated time-points (*n* = 4–8 mice per group). **(B)** Forebrain *in vivo* glial activation assessed by 18-kDA translocator protein (TSPO) [^18^F]-GE180 μPET is presented as standard-uptake-value-ratios (SUVR) relative to white matter reference tissue uptake (*n* = 8 mice per group). **(C)** *Post mortem* levels of total β-amyloid peptide (Aβ) were quantified by ELISA from forebrain tissue at indicated time-points (*n* = 4–8 mice per group). **(D)** Forebrain *in vivo* amyloidosis assessed by [^18^F]-florbetaben μPET is presented as SUVR relative to white matter reference tissue uptake (*n* = 8 mice per group). Data information: in **(A–D)**, data are presented as mean ± SD. *significant differences in PS2APP mice vs. their 5 month old littermates (*p < 0.05; **p < 0.001); ^#^significant differences in PS2APP mice vs. age-matched wild-type (WT; ^#^*p* < 0.05; ^##^*p* < 0.001); °significant differences between young and aged WT (°*p* < 0.05). Analysis of variance (ANOVA) with Tukey *post hoc* applies for all. Please note that data of **(B,D)** were partially previously published (Brendel et al., 2016).

Two independent studies have recently shown a significant increase of CSF sTREM2 as a function of age (Piccio et al., 2016; Suárez-Calvet et al., 2016b). Furthermore, transgenic amyloid mouse models indicate an age dependent increase of TREM2 mRNA expression in the brain (Matarin et al., 2015). Hence, we first investigated the levels of sTREM2 in *post mortem* brain homogenates of PS2APP mice (Richards et al., 2003; Ozmen et al., 2009) and WT littermates at different ages. ELISA based quantification of sTREM2 in TBS extracts showed a significant age-dependent increase of sTREM2 in the non-transgenic C57BL/6 mice (Figure 1A; +19.2%; *p* < 0.05), which is in line with an age-dependent increase of sTREM2 seen in cognitively normal humans (Suárez-Calvet et al., 2016b). Similarly, the TSPO μPET signal also showed a small but significant increase in aged WT mice (Figure 1B; +4.0%; *p* < 0.05).

In line with our previous μPET findings of increased fibrillar amyloid deposition and glial activation in PS2APP mice with age (Brendel et al., 2016), we observed a significant progressive increase of sTREM2 (5 vs. 8 months: +82%, *p* < 0.05; 5 vs. 13 months: +165%, *p* < 0.001; 5 vs. 16 months: +211%, *p* < 0.001; Figure 1A) which was far in excess of the age-dependent increases in WT animals. Quantitation of the proinflammatory cytokines Interleukin 1b, Interleukin 6 and KC/GRO (Interleukin 8 related protein) as well showed age-dependent increases in PS2APP, however to a lesser extent compared to sTREM2.

In parallel, the TSPO μPET signal indicated the known increase with age across the lifespan of PS2APP mice (Figure 1B). Total Aβ advanced with age at similar extent when compared to sTREM2 (5 vs. 8 months: +59%, p = n.s.; 5 vs. 13 months: +165%, *p* < 0.001; 5 vs. 16 months: +223%, *p* < 0.001; Figure 1C). Fibrillary plaque signal assessed by Aβ μPET showed a delayed increase in the entire forebrain from 8 months onwards (Figure 1D).

**Figure 2:**
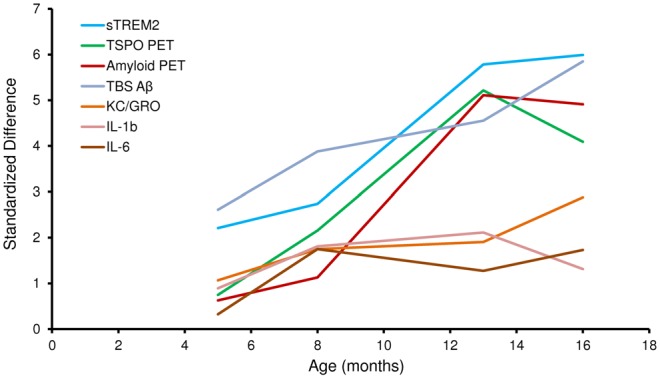
**Standardized differences for biochemical and imaging modalities during the life course of PS2APP mice.** Data information: standardized differences between PS2APP and WT mice were computed by the delta divided by the standard deviation for all assessment points in PS2APP. The development of measures in WT was assumed linear when no direct comparison time-point was available.

Figure 2 illustrates the standardized differences of biochemical and imaging measures for the PS2APP mouse model during their life course. For this purpose, age dependent differences between PS2APP and WT mice were computed (under the assumption that measures in WT with only two time-points are developing linearly during aging) and divided by the standard deviation in PS2APP mice. Here standardized differences of TSPO-PET and sTREM2 as well reached or even exceeded the level of fibrillar Aβ deposition and total Aβ. Standardized sTREM2 differences clearly exceeded those of common cytokines at all time points. In summary, the standardized timelines of sTREM2, TSPO-PET, Aβ-PET and total Aβ were characterized by a high concordance.

Together, these results suggest that microglia activation, as measured by TSPO μPET signal and sTREM2 measurement, increase with normal aging, and this increase is strongly augmented by amyloid pathology.

### sTREM2 Is Highly Correlated with *In Vivo* TSPO and Amyloidosis Biomarkers in PS2APP Mice

**Figure 3:**
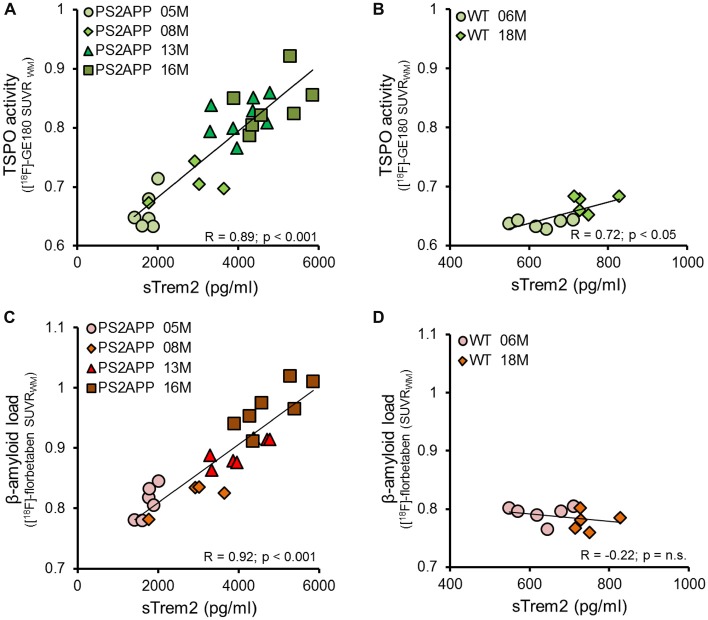
**sTREM2 levels strongly correlate with dual tracer μPET *in vivo* measurements of TSPO and amyloidosis. (A,B)** Correlation of *post mortem* levels of sTREM2 in the forebrain with *in vivo* TSPO [^18^F]-GE180 μPET measurements in PS2APP and WT mice. **(C,D)** Correlation of *post mortem* levels of sTREM2 in the forebrain with *in vivo* amyloid [^18^F]-florbetaben μPET measurements in PS2APP and WT mice. Data information: for all data Pearson’s coefficient of correlation (R) was calculated together with the significance level using SPSS.

Given the age-dependent increases of the several biomarkers, we subsequently assessed the correlation between sTREM2 levels and μPET results. The concentration of sTREM2 in the brain showed a strong correlation with the TSPO μPET signal in PS2APP mice (*R* = 0.89, *p* < 0.001; Figure 3A). The same analysis in WT mice also indicated a positive correlation, although less pronounced (*R* = 0.72, *p* < 0.05; Figure 3B). A strong positive correlation was as well observed between sTREM2 level and fibrillar amyloidosis as assessed by Aβ μPET in PS2APP (*R* = 0.92, *p* < 0.001; Figure 3C) but not in WT mice (Figure 3D). The correlation between total Aβ and sTREM2 levels (*R* = 0.95; *p* < 0.001) further supported the strong association between sTREM2 and amyloidosis.

Next, we asked whether brain levels of sTREM2 are associated with TSPO and Aβ μPET signals independently from age. To this end, we performed a multiple regression analysis with sTREM2 as an outcome variable, with TSPO μPET and Aβ μPET as predictors, and with introduction of age as a covariate. In this analysis, forebrain TSPO μPET signal was significantly associated with sTREM2 (*β* = 0.40; *p* < 0.05), after adjusting for age; forebrain TSPO μPET signal and age together accounted for 84% of the variance in sTREM2 (*F*_(2,22)_ = 61.5, *p* < 0.0001, *R*^2^ = 0.85, *R*^2^_Adjusted_ = 0.84). Forebrain Aβ μPET signal was also significantly associated with sTREM2 (*β* = 0.53; *p* < 0.01), after adjusting for age; forebrain Aβ μPET signal and age together accounted for 86% of the variance in sTREM2 (*F*_(2,22)_ = 77.5, *p* < 0.0001, *R*^2^ = 0.88, *R*^2^_Adjusted_ = 0.86).

In summary, we found a strong correlation of sTREM2 levels and μPET findings of microglial activation and fibrillar amyloidosis in PS2APP mice, a correlation that was highly driven by age. Nonetheless, significant associations between sTREM2 levels and uptake of both μPET tracers still remained, even upon exclusion of age as a nuisance variable.

### Widespread TSPO Activation Is Already Established in Young PS2APP Mice

In the second step of image analysis, we used voxel-wise dual tracer μPET results for a more detailed mapping of the sequence of neuropathological alterations in the PS2APP mice. In particular, we used SPM to test for spatial and temporal relationships between neuroinflammation and amyloidosis as measured by μPET in transgenic vs. age-matched WT mice. Additional histochemical analyses were undertaken in order to support the detection of a very early stage of fibrillar amyloid deposition in the younger PS2APP mice, where detection thresholds of μPET might be too low to detect the presence of early pathology.

**Figure 4:**
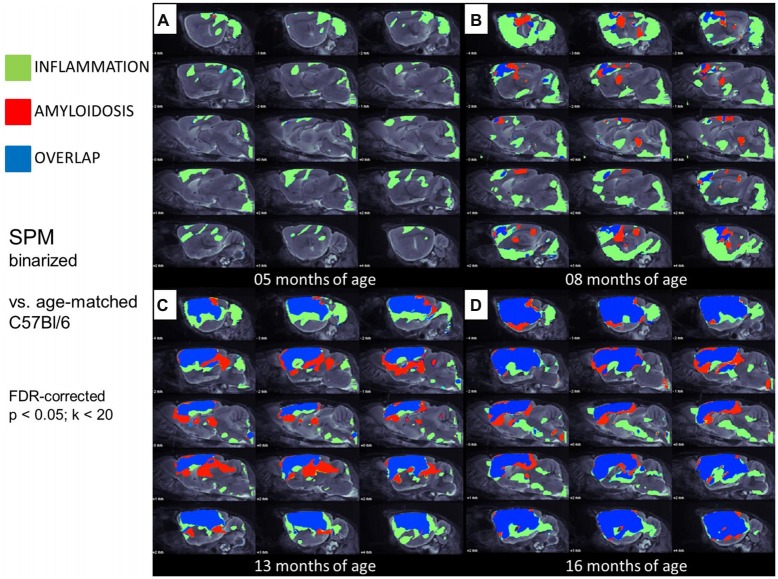
**Glial activation and fibrillar amyloidosis progress regionally concerted during the life-cycle of PS2APP mice. (A–D)** Voxel-wise regional distribution of elevated *in vivo* radiotracer uptake in PS2APP mice compared to WT at all four different age stages. Increases in TSPO [^18^F]-GE180 μPET binding, indicating glial activation (green), increases in amyloid [^18^F]-florbetaben μPET binding (red), and areas of overlapping increases for both radiotracers (blue) are projected upon sagittal slices of an MRI mouse atlas. Data information: in **(A–D)** all voxels of the mouse brain were compared by a student’s *t*-test in SPM. All significant differences exceeding a threshold of *p* < 0.05, including FDR-correction for multiple comparisons were binarized for each tracer.

**Figure 5:**
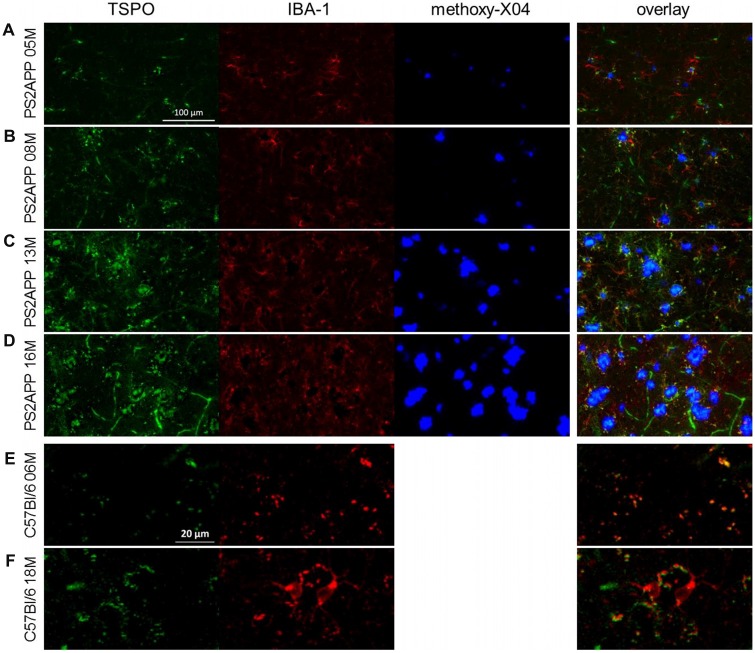
**Immunohistochemistry confirms μPET findings. (A–D)** Immunohistochemistry in the frontal cortex of PS2APP and WT mice at different ages. TSPO staining is depicted in green. IBA-1 positive cells are illustrated in red. Fibrillar Aβ plaques stained by methoxy-X04 are shown in blue. Scale bar in **(A)** indicates 100 μm. **(E,F)** Immunohistochemistry in the frontal cortex of WT mice at different ages. TSPO staining is depicted in green. IBA-1 positive cells are illustrated in red. Please note that anti-TSPO recognizes an antigen in blood vessels a phenomenon that is well described in the literature. Therefore a higher zoom was used for a proper visualization of increasing IBA-1 positive microglia in aged WT. Scale bar in **(E)** indicates 20 μm.

In this analysis, TSPO μPET revealed relevant microglial activation (11% of the total brain volume; *p* < 0.05, FDR-corrected) in PS2APP mice as young as 5 months, with main foci in the frontal and parietal cortices, and also in the cerebellar hemispheres (Figure 4A). At this early age, punctate fibrillar amyloid depositions were detected by methoxy-X04 histology in the frontal cortex of all PS2APP mice (Figure 5A), but these were not sufficiently pronounced to impart a [^18^F]-florbetaben Aβ μPET signal robust to correction for multiple comparisons.

At 8 months of age, nearly one third of the brain volume in PS2APP mice had increased TSPO μPET signal (32%; *p* < 0.05, FDR-corrected; Figure 4B). Most of the regions with new TSPO μPET specific binding arising between 5 and 8 months were in the frontal, piriform and entorhinal cortical areas. 7% of the brain volume had significantly elevated Aβ μPET signal at 8 months, with foci mainly in the frontal cortex and thalamus (*p* < 0.05, FDR-corrected; Figure 4B), of which total 3% of the total brain volume corresponded to regions with overlapping TSPO and fibrillar Aβ increases relative to corresponding μPET results in WT mice. Immunohistochemical analysis in the same animals showed numerous small fibrillar plaques, generally matching the regions with elevated amyloid μPET signal, and to microscopic examination, surrounding IBA-1 and TSPO positive cells (Figure 5B).

Together, these results indicate that microglial activation is already established in large parts of the brain in young PS2APP (5 and 8 months) mice, whereas fibrillar Aβ deposition is less pronounced and restricted to specific areas. Inflammatory processes clearly surround the establishment of early plaques, but occur as well in areas without manifest fibrillar Aβ deposition in young PS2APP mice, in which over-expressed Aβ is probably present only in soluble form.

### TSPO Activity and Amyloidosis Progress Concerted in Aging PS2APP Mice

Next, we mapped the regional progression of TSPO and Aβ μPET signals in PS2APP mice aged more than 8 months in order to discern the spatial relationship of the two biomarkers later in life. We noted the enlargement of areas with overlapping increases detected by dual tracer μPET in the forebrain, where the total brain volume of concerted microglial activation and amyloidosis increased from only 3% at 8 months to 24% at 13 months and 37% at 16 months (Figures 4B–D). Thus, more than one third of the entire brain volume, notably in the forebrain cortices, thalamus and hippocampus, manifested concerted microglial activation and amyloidosis at 16 months of age. This result was further strengthened by the increase in dice similarity coefficients from initially low levels (1% similarity at 5 months and 12% similarity at 8 months), progressing to scores indicating very high agreement between brain voxels with elevated TSPO and Aβ μPET binding in aged PS2APP mice (63% similarity at 13 months and 69% similarity at 16 months of age).

Immunohistochemical analyses confirmed the μPET findings and indicated an age-dependent increase of fibrillary Aβ plaques surrounded by numerous IBA-1 and TSPO positive cells (Figures 5C,D). The WT mice themselves indicated an age-dependent increase of IBA-1 positive cells (Figures 5E,F), confirming the findings of a slight age-dependent elevation of TSPO μPET signal together with increasing sTREM2 levels in aged WT mice (Figures 1A, 3B).

We conclude from these voxel-wise dual tracer μPET results that TSPO activity and fibrillar amyloidosis progress in a concerted manner in PS2APP mice aged more than 1 year, ultimately co-expressing in most of the forebrain. In contrast, WT mice showed only a subtle age-dependent neuroinflammation, as revealed by IBA-1 positive cells by immunohistochemistry. This result in WT mice confirmed the significant increases in TSPO μPET signal between 6 and 18 months of age.

## Discussion

We present the first study employing *post mortem* assessment of sTREM2 and Aβ in conjunction with dual-tracer small animal μPET for TSPO and Aβ, aiming to reveal the spatial and temporal relation between microglial activation and amyloidosis during the life-course of a mouse AD model. The main findings of the present study are: (1) sTREM2, TSPO and amyloidosis progressively increase with age in the PS2APP mouse model of AD; (2) sTREM2 levels, TSPO μPET and Aβ μPET uptake are all strongly associated with each other, and still after exclusion of age; and (3) there is widespread microgliosis in young PS2APP mice, along with early punctate fibrillar Aβ deposition, which progresses to extensive overlap of microglial activation and amyloidosis in the 16 month old mice.

Our results show that sTREM2 levels and TSPO μPET signal increase in parallel during normal aging of WT mice, but that increase is more pronounced when there is underlying amyloid pathology. These preclinical results are consistent with findings previously described in humans in a study showing an association of CSF sTREM2 levels with increasing age both in cognitively normal subjects, and in AD patients at different disease stages (Suárez-Calvet et al., 2016b). Importantly, the increase of CSF sTREM2 as a function of age was more pronounced in patients with underlying AD pathology (even if they were in a preclinical stage) than in cognitively normal subjects, a phenomenon which is mirrored by the amyloid mouse model used in this study. It is difficult to make a direct quantitative comparison of Aβ deposition between human and mouse PET data. However, the standardized differences of biochemical and imaging measures for the PS2APP mouse model illustrated in Figure 2 show a >5-fold in the double transgenic PS2APP mice for Aβ-PET, as compared by standardized differences <2-fold in patients with dominantly inherited AD (Bateman et al., 2012), which gives a rough impression of the heavy amyloidosis in PS2APP mice. Moreover standardized differences of TSPO-PET and sTREM2 reached or even exceeded the level of fibrillar Aβ deposition and total Aβ, underlining the parallel increase of neuroinflammation and amyloidosis with age in the studied mouse model.

In contrast, CSF sTREM2 samples of human dominantly inherited AD increase below and temporally after Aβ, where sTREM2 levels peak at symptom onset and drop again in the later disease course (Suárez-Calvet et al., 2016a). In sporadic AD the highest levels of CSF sTREM2 are as well observed in early pre-symptomatic stages and likewise decrease in manifest AD (Suárez-Calvet et al., 2016b). In opposite our mouse model is characterized by a linear increase of sTREM2 with advancing age. We attribute the current findings to the fact that the mouse model—with ongoing amyloid deposition but very low levels of neuronal death—most likely resembles the pre-symptomatic stage in humans. Noteworthy, highest absolute deposition rates of Aβ by longitudinal PET are observed at presymptomatic and MCI stages of sporadic human AD (Villemagne et al., 2013; Brendel et al., 2015a). Additionally CSF sTREM2 and Aβ only indicate an association at the MCI stage of sporadic AD (Suárez-Calvet et al., 2016b). Taken together sTREM2 might be specifically elevated during active amyloid deposition in mice and humans. Such considerations are in line with the time-dependent increase of TREM2 mRNA in other Aβ transgenic mouse models (Matarin et al., 2015; Wang et al., 2015), as well as an initial report of increased plaque-associated TREM2 expression (Frank et al., 2008).

Thus, our results demonstrate that amyloid pathology is able to trigger microglial activation, irrespective of effect of neurofibrillary tangles on neurodegeneration and neuronal death *per se*. These results do not necessary contradict the increasing evidence that TREM2 recognizes apoptotic neurons and mediates their phagocytosis (Wang et al., 2015), and likewise the fact that CSF sTREM2 are highly associated with markers of neural injury (T-tau) and neurofibrillary tangle neurodegeneration (P-tau; Heslegrave et al., 2016; Piccio et al., 2016; Suárez-Calvet et al., 2016b). Instead, these results speak to the possibility that both amyloid pathology and neuronal death trigger microglial activation and TREM2 production.

A key novelty of this study is due to the dual *in vivo* imaging of neuroinflammation and fibrillar amyloidosis, which preceded the assessment of TREM2 *post mortem*. The results of TSPO imaging *in vivo* are strengthened by recent investigations in transgenic AD mouse models: [11C]PBR28 showed a 27% increase of brain uptake in 6 month old 5XFAD mice compared to WT mice, confirmed by autoradiograpy and immunohistochemistry (Mirzaei et al., 2016). Another study in which the current TSPO tracer was successfully applied revealed age-dependent increase of neuroinflammation in APP/PS1dE9 mice, as confirmed by autoradiography *ex vivo* and immunohistochemical *in vitro* assessments (Liu et al., 2015). Additionally, our own validation study of the TSPO radioligand proved that its kinetics favor robust and reproducible TSPO imaging in PS2APP and WT mice, and gave high agreement with *post mortem* immunohistochemistry findings (Brendel et al., 2016).

In general, μPET can serve for longitudinal monitoring ahead of terminal readouts in interventional designs, where therapeutically induced alterations in biomarker associations can be followed. Such studies addressing the effect of genetic or therapeutic inhibition of microglial activation (including TREM2) on amyloid deposition rates are the focus of ongoing research (Birch et al., 2014; Tanzi, 2015). Furthermore, knowing the individual baseline pathology levels at therapy initiation, allows for more sensitive detection of longitudinal biomarker changes in individual mice, as shown in our recent anti-amyloid intervention μPET study (Brendel et al., 2015c). Besides facilitating therapeutic advances, the advantage of longitudinal assessments is also highlighted by a recent study hypothesizing that different timing of analyses accounts for discrepant findings of the effects of TREM2 deficiency on Aβ accumulation (Wang et al., 2016). By dual μPET longitudinal assessment, we can reveal dynamic Aβ and TSPO phenotypic changes over time, which inform the interpretation of terminal readouts, and may illuminate pathogenic mechanisms.

**Figure 6:**
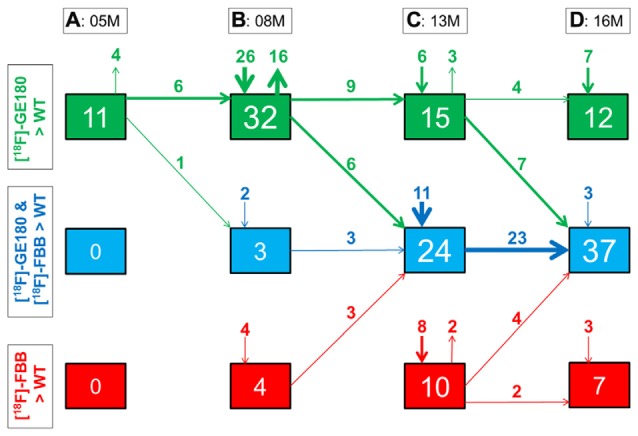
**Quantitative progression of glial activation and fibrillar amyloidosis during the life-cycle of PS2APP mice. (A–D)** Percentage (%) of brain voxels relative to the whole brain in which binding of either [^18^F]-GE180 (green) or [^18^F]-florbetaben (FBB, red) or both tracers (blue) in groups of PS2APP mice exceeded that in WT mice at 5, 8, 13 or 16 months of age. Data information: arrows between boxes indicate the propagation of changing volume percentages for the two markers between groups of increasing age. Inward and outward arrows indicate the amount volume percentage of newly presenting/disappearing voxels for the markers at each specific age group. Thin arrows indicate 1–5% volume changes, medium arrows 5–10% changes, and thick arrow show changes exceeding 10% volumes. In **(A–D)** all voxels of the mouse brain were compared by a student’s *t*-test in statistical parametric mapping (SPM). All significant differences exceeding a threshold of *p* < 0.05, including FDR-correction for multiple comparisons were binarized for each tracer.

Our regional μPET analyses allowed a detailed mapping of TSPO activation and fibrillar amyloidosis in the mouse brain over time. We find that young PS2APP mice already manifest wide-spread microglial activation, even at an age when fibrillary amyloid deposition is sparse, and only detectable by highly sensitive histological methods, rather than by the less sensitive [^18^F]-florbetaben μPET method, which yielded SUVR not significantly elevated compared to common background ratios observed in WT mice. This finding is in line with another preclinical study that showed astrocytosis to precede fibrillar amyloidosis in APPswe mice at 6 months of age (Rodriguez-Vieitez et al., 2015). As total Aβ levels in our study were already elevated at 5 months of age (Figure 1B), it seems likely that soluble Aβ components, i.e., monomers and oligomers, and the highly overexpressed APP itself may together trigger early neuroinflammatory processes (Minter et al., 2015). Temporal regional results for both radiotracers support this assumption, as TSPO activity was elevated in quantitatively extensive brain regions still lacking fibrillar amyloid plaques at 5 and 8 months of age (Figure 6).

The study has some limitations. First, we used μPET data derived from a cross-sectional study, since a true longitudinal design would not easily have afforded longitudinal sTREM2 assays; we expect that repeated CSF sampling would have greatly increased dropout rates. Overall, we feel that biases possibly arising from the cross-sectional design are well balanced by the use of adequately powered group sizes. In the immunohistochemistry end readout we focused on well-established IBA-1 staining and robust TSPO staining. However, vessel staining by TSPO antibodies still hampering quantitation, and may imperil our conclusions about group differences. We consider it unlikely that TSPO vessel staining contributes importantly to the TSPO μPET signal, since 6 month old WT mice with heavy vessel staining had significantly lower [^18^F]-GE180 SUVR when compared to PS2APP mice at 5 months. Thus, we consider the vessel staining in immunohistochemistry as likely to be unspecific. Increased levels of sTREM2 in the used double transgenic mouse model may be influenced by altered γ-secretase activity (Wunderlich et al., 2013). Although we deem strong effects to be unlikely, it will be valuable to confirm current findings in AD models without overexpression of PSEN or APP.

## Conclusion

In conclusion, PS2APP mice show a strong correlation between sTREM2 levels, microglial activation and amyloidosis, indicating that sTREM2 serves as a biomarker for microglial activity, which is, in turn, highly driven by the heavy amyloidosis in this mouse model. Our data suggest that sTREM2 may increase during active amyloid deposition and that the inflammatory response in this mouse model possibly reflects a specific stage of AD, most likely the preclinical stage, characterized by high amyloid accumulation rates. Regional data indicate widespread microglial activation in young PS2APP mice, and concerted progression of amyloid pathology and TSPO elevation during their life course. Causal relationships need to be tested in longitudinal multi-tracer μPET studies with interventions targeting specific aspects of AD-like pathology.

## Author Contributions

MB participated in the design of the study, performed the data analysis, and drafted the manuscript. GK participated in the design of the study, contributed to global data analysis, performed biochemical assessments and drafted the manuscript. FP and FO carried out the PET experiments and performed PET data analysis. AJ and TB carried out the histological experiments, performed the histological data analysis, and drafted the manuscript. NLA helped to draft the manuscript. JC participated in the PET experiments and helped to draft the manuscript. SL and FJG carried out the radiochemistry. LO, KB and PB participated in the design of the study and helped to draft the manuscript. MS-C, ME and CH participated in the design of the study and increased the intellectual content. JH participated in its design and coordination and drafted the manuscript. AR conceived of the study, and participated in its design and coordination, contributed to interpretation of the data and drafted the manuscript. All authors read and approved the final manuscript.

## Funding

The study was financially supported by the SyNergy Cluster (JH, PB, CH and AR) and by the European Research Council under the European Union’s Seventh Framework Program (FP7/2007–2013)/ERC Grant Agreement No. 321366-Amyloid (advanced grant to CH). AJ was supported by the Foundation for Polish Science within the International PhD Project “Studies of nucleic acids and proteins—from basic to applied research”, co-financed from European Union—Regional Development Fund; MPD/2009-3/2. Florbetaben precursor was provided by Piramal Imaging. GE180 cassettes were received from GE.

## Conflict of Interest Statement

LO and KB are employees of F. Hoffmann-La Roche; PB received consultant fees from GE and Piramal Imaging, and honoraria from Siemens; AR received consultant fees from Piramal Imaging and GE. The remaining authors declare they have no conflict of interest.

## Abbreviations

AD: Alzheimer’s disease
APP: amyloid precursor protein
ANOVA: Univariate analysis of variance
Aβ: β-amyloid peptide
BCA: bicinchoninic acid
CSF: cerebrospinal fluid
PS: presenilin
SPM: statistical parametric mapping
sTREM2: soluble triggering receptor expressed on myeloid cells 2
SUVR: standard-uptake-value-ratios
TBS: Tris-buffered saline
TREM2: triggering receptor expressed on myeloid cells 2
TSPO: 18-kDA translocator protein
TX_rigid_: rigid-body transformation
TX_BN_: non-linear brain normalization
VOI: volume-of-interest.
